# Proteome Analysis of Watery Saliva Secreted by Green Rice Leafhopper, *Nephotettix cincticeps*

**DOI:** 10.1371/journal.pone.0123671

**Published:** 2015-04-24

**Authors:** Makoto Hattori, Setsuko Komatsu, Hiroaki Noda, Yukiko Matsumoto

**Affiliations:** 1 National Institute of Agrobiological Sciences, 1-2 Ohwashi, Tsukuba, Ibaraki, 305-8634, Japan; 2 National Institute of Crop Science, 2-1-18 Kannondai, Tsukuba, Ibaraki, 305-8518, Japan; University College Dublin, IRELAND

## Abstract

The green rice leafhopper, *Nephotettix cincticeps*, is a vascular bundle feeder that discharges watery and gelling saliva during the feeding process. To understand the potential functions of saliva for successful and safe feeding on host plants, we analyzed the complexity of proteinaceous components in the watery saliva of *N*. *cincticeps*. Salivary proteins were collected from a sucrose diet that adult leafhoppers had fed on through a membrane of stretched parafilm. Protein concentrates were separated using SDS-PAGE under reducing and non-reducing conditions. Six proteins were identified by a gas-phase protein sequencer and two proteins were identified using LC-MS/MS analysis with reference to expressed sequence tag (EST) databases of this species. Full -length cDNAs encoding these major proteins were obtained by rapid amplification of cDNA ends-PCR (RACE-PCR) and degenerate PCR. Furthermore, gel-free proteome analysis that was performed to cover the broad range of salivary proteins with reference to the latest RNA-sequencing data from the salivary gland of *N*. *cincticeps*, yielded 63 additional protein species. Out of 71 novel proteins identified from the watery saliva, about 60 % of those were enzymes or other functional proteins, including GH5 cellulase, transferrin, carbonic anhydrases, aminopeptidase, regucalcin, and apolipoprotein. The remaining proteins appeared to be unique and species- specific. This is the first study to identify and characterize the proteins in watery saliva of Auchenorrhyncha species, especially sheath-producing, vascular bundle-feeders.

## Introduction

Most species of hemipterans, including vascular bundle feeders, produce gelling and watery saliva during the feeding process [1–3]. When these insects probe into plant tissues with their stylets, they discharge a viscous mixture of saliva, which coagulates and forms into a feeding mark (salivary flange) and salivary sheaths. [4–6]. It has been reported that aphids secrete watery saliva from the onset of penetration into the plant tissue [7]. Thus, saliva is involved from the beginning of insect-plant encounter, and is considered to contain some molecules that modulate, evade, or suppress plant defense, enabling the insect to feed on sap safely and successfully [6]. Nevertheless, some other molecules found in saliva induce plant defense response [8]. Therefore, the saliva may play a crucial role in determining the compatibility between the insect and the plant.

The majority of information regarding the salivary components of phytophagous insects is for aphids, which ingest sap from vascular bundles, mainly phloem. Initially, the presence of enzymes such as oxidases and hydrolases in the saliva, was confirmed in agarose-substrate or sucrose solutions that aphids had fed upon by visualizing or detecting their enzymatic reactions from the resulting appearance of color [9–12]. Subsequently, salivary proteins collected from large amounts of fed-upon diet were separated electrophoretically and the N-terminal amino acid sequences were determined for the major proteins in *Schizaphis graminum* [13]. Related proteins sharing common properties were identified in the saliva of other aphid species such as *Acyrthosiphon pisum* and *Myzus persicae* using the antibodies of *S*. *graminum* saliva [14]. Recently, the availability of aphid genome and transcriptome sequences enables direct identification of various salivary proteins from several aphid species [15–21]. These studies revealed that some of the major components, such as glucose dehydrogenases, carbonic anhydrases, and proteases are ubiquitous in the saliva of many aphid species. Additionally, transcriptome and proteome analysis of salivary glands in hemipterans helped create a comprehensive catalog of all secretory proteins in this tissue [22–24]; however, it is difficult to differentiate the actual salivary components from secretory proteins of the salivary glands.

There have been only limited studies on the secreted saliva of phytophagous hemipteran species other than aphids. The green rice leafhopper, *Nephotettix cincticeps* (Uhler), is a major insect pest of rice and in Japan and East Asian countries that causes damages through direct feeding or transmitting virus and phytoplasma pathogens [25–27]. Leafhoppers primarily feed on the vascular bundles of rice and other Poaceae host plants, imbibing the contents of phloem sieve tubes, the xylem, and rarely the mesophyll cells [28]. Like most other hemipterans, during the feeding process, it discharges gelling and watery saliva, which contain various components, including enzymes, such as laccase-1 and β-glucosidase [3, 29–31], and a Ca^2+^-binding protein [32]. However, the total composition of the salivary cocktail and its potential functions for successful feeding from plants remains unknown to date.

In this study, we analyzed the proteineous components in the watery saliva of *N*. *cincticeps* in order to identify and characterize the molecules that have potential functions in the feeding process. Major bands of salivary proteins were separated by polyacrylamide gel electrophoresis (SDS-PAGE) and subjected to N-terminal sequencing followed by molecular cloning. N-terminally blocked proteins were analyzed utilizing *N*. *cincticeps* expressed sequence tag (EST) database for protein identification. In addition, many other minor proteins in the watery saliva were identified using gel-free based nano liquid chromatography (LC)-tandem mass spectrometry (MS) analysis, as we have most recently completed RNA-sequencing (RNA-seq) of salivary gland transcriptome in *N*. *cincticeps* [33].

## Materials and Methods

### Collection of Insects and Salivary Proteins

We collected *N*. *cincticeps* in an experiment paddy field of Japan International Research Center for Agriculture Sciences in Tsukuba, Ibaraki, Japan in 1993. Insects were maintained on rice seedlings (var. Koshihikari) at the National institute of Agrobiological Sciences. No specific permission is required for capturing of this insect pest. We caught live insects with an insect net in the paddy field, but released all insects collected except *N*. *cincticeps*.

One ml of a 5% (w/v) sucrose diet was sealed between two layers of stretched Parafilm and attached to each of 50-mm petri dish with a piece of filter paper in the bottom. About 15 adult leafhoppers were allowed to feed in each dish at 25°C for 10 hours [32]. The fed-diet solution containing secreted saliva (ca. 0.76 ml per dish) was pooled, filtrated with 1.2 μm Minisalt filter (Sartorius Stedium, Goettingen, Germany) and ultra-filtered with a 10,000 MWCO AmiconUltra-15 filter (Millipore, Billerica, MA, USA). To obtain one μg of the salivary proteins, 40 to 50 adults were required. The protein samples used for different analyses were independently collected.

### N-terminal Amino Acid Sequence Analysis of Major Salivary Components of Saliva and Database Search

We analyzed saliva samples using SDS-PAGE, since 2-dimensional electrophoresis has a number of limitations, including the difficulty to separate very large or very small proteins and acidic or basic proteins [34]. Ultrafiltrated salivary proteins (ca. 150 μg) were separated using 12.5% SDS—PAGE (138 mm × 130 mm) under reducing conditions. A sample buffer consisting of 0.25M Tris-HCl (pH 6.8), 20% glycerol, 5% SDS, and 0.4 M dithiothreitol (DTT) was added to the protein solution, and heated at 95°C for 5 min before electrophoresis. Another sample (ca. 45 μg) was loaded onto a 15% pre-cast e-PAGEL (ATTO Co., Tokyo, Japan) (90 mm × 83 mm) in a Tris-tricine buffer under non-reducing conditions. Protein solution was combined with the sample buffer, but without DTT or heating. Each gel was electro-blotted on a polyvinylidene difluoride (PVDF) membrane using an SDS-PAGE with Trans-Blot SD semi-dry transfer cell (Bio-Rad, Hercules, CA, USA) or an e-PAGEl with Mini Trans- Blot Electrophoretic Transfer Cell (Bio-Rad, Hercules, CA, USA). Protein bands were stained with Coomassie brilliant blue (CBB) and subjected to N-terminal amino acid sequencing using PROCISE cLC protein sequencing system (Applied Biosystems, Foster City, CA, USA). N-terminal amino acid sequences were searched using translated nucleotide database (TBLASTn) against EST database of *N*. *cincticeps*. EST/cDNA libraries of *N*. *cincticeps* consisting of 25,061 sequences were constructed from different tissues, including salivary glands (8436), midgut (5908), mycetome (3095), ovary (4364), and testis (3258) (Noda, unpublished).

### LC-MS/MS Analysis of N-terminal Blocked or Minor Salivary Proteins and Database Search

Proteins with blocked N-termini and low abundance proteins were analyzed using LC-tandem MS as described previously [35], with minor modifications. In brief, concentrated salivary proteins (ca. 580 μg) were separated on 13.5% SDS—PAGE (138 mm × 130 mm) under reducing conditions and stained with CBB. Protein gel bands were washed with 25% methanol and 7% acetic acid for 12 h, and destained with 50 mM NH_4_HCO_3_ in 50% methanol for 1 h at 40°C. Proteins were reduced with 10 mM DTT in 100 mM NH_4_HCO_3_ and alkylated with 40 mM iodoacetamide in 100 mM NH_4_HCO_3_. Proteins were treated with 1 pM trypsin in 100 mM NH_4_HCO_3_ overnight at 37°C. Digested peptides extracted with 30 μl of 0.1% trifluoroacetic acid in 50% acetonitrile/water were desalted with NuTip C-18 pipette tips (Glygen, Columbia, MD, USA), and characterized by electrospray ionization (ESI) MS and MS/MS utilizing a quadrupole time-of-flight (Q-TOF) MS (Micromass, Manchester, UK). Mass spectra were searched against a database constituted from ESTs of *N*. *cincticeps* described above using MASCOT software (Matrix Science, London, UK).

### Identification of Minor Salivary Proteins Using Nano-liquid Chromatography—Tandem MS Analysis

Since differences between the proteins identified from in-gel and gel-free approaches have been reported [36], saliva samples were analyzed using gel-free based LC-MS/MS [37] with reference to the latest RNA-sequencing data from salivary gland of *N*. *cincticeps* [33]. Proteins in the samples (46 μg/120 μL) were reduced with 10 mM DTT for 1 h at 37°C and alkylated with 20 mM iodoacetamide for 1 h min at 37°C in the dark. Alkylated proteins were digested with trypsin and lysyl endopeptidase (Wako, Osaka, Japan) at 1:100 enzyme/protein concentrations at 37°C for 16 h. The peptides were dried and tryptic peptides were acidified to pH<3 with 10 μL of 20% formic acid, which were desalted with NuTip C-18 pipet tip. Two μL (< 9 μg) were subjected to nanospray LTQ XL Orbitrap MS (Thermo Fisher Scienific, San Jose, CA, USA). Peptides in 0.1% formic acid were loaded onto an Acclaim PepMap300 C18 column sets (Dionex, Germering, Germany) using an UltiMate 3000 Nano LC (Dionex). Peptides eluted from the trap column were separated and sprayed using 0.1% formic acid in acetonitrile at a flow rate of 200 nL min^-1^ on a C18 Tip column (75 μm ID × 120 mm, nano HPLC capillary column, NTTC-360/75-3; Nikkyo Technos, Tokyo, Japan) with a spray voltage of 1.8 kV. Elution was performed using a linear acetonitrile gradient (10–30% in 90 min) in 0.1% formic acid. Full-scan mass spectra were acquired in the Orbitrap over a mass range of 400–1,500 m/z with a resolution of 30,000. The six most intense precursor ions were subjected to collision-induced fragmentation in the linear ion trap at normalized collision energy of 35% after accumulation of a target value of 1,000. Dynamic exclusion was employed within 90 sec to prevent repetitive selection of peptides. MS/MS spectra were analyzed using MASCOT software using *N*. *cincticeps* salivary gland RNA-seq data with 51,788 contigs (DDBJ Bio-Project Database ID PRJDB1562, [33]) available at ftp://ftp.ddbj.nig.ac.jp/ddbj_database/dra/fastq/DRA002/DRA002228/DRZ003173/provisional/). The acquired raw data files were processed using Proteome Discoverer (version 1.4, Thermo Fisher Scientific), and the parameters used in Mascot searches were as follows: carbamidomethylation of cysteine was set as a fixed modification and oxidation of methionine was set as a variable modification. Trypsin was specified as the proteolytic enzyme and one missed cleavage was allowed. Peptide mass tolerance was set at 5 ppm, fragment mass tolerance at 0.5 Da, and peptide charges at +2, +3, and +4. An automatic decoy database search was performed as part of the search. The mass spectrometry proteomics data have been deposited with the ProteomeXchange Consortium (http://proteomecentral.proteomexchange.org) via the Proteomics identifications Database (PRIDE) partner repository [38] with the dataset identifier PXD001785.

Peptides with a percolator ion score of more than 13 (p < 0.05) were used, and the number of matched unique peptide (more than 2) was considered to identify proteins.

### 5′- and 3′-RACE and Sequence Analysis of Major Salivary Proteins

EST sequences were assembled into contigs and were extended by 5′ and 3′ RACE-PCR, in order to obtain full-length cDNA of major salivary proteins (S1 Table, primers) using SMARTer RACE cDNA Amplification Kit (Clontech, Mountain View, CA, USA), when needed, following manufacturer's instructions. When a protein did not correspond to any EST sequences in the database, its gene was amplified using degenerate primers based on its N-terminal sequence (S1 Table). Nucleotide and deduced amino acid sequences of the major salivary proteins were analyzed using Basic Local Alignment Search Tool (BLAST). Predictions of signal peptide cleavage sites and glycosylation site usage were carried out with SignalP, NetNGlyc, and NetOGlyc on CBS Prediction Server (http://www.cbs.dtu.dk/services/). Putative transmembrane domains (hydrophobicity sites) were predicted using SOSUI (http://bp.nuap.nagoya-u.ac.jp/sosui/) [39]. Furthermore, biological information related to protein function, sequence motifs, and structural domains was acquired using InterProScan (http://www.ebi.ac.uk/interpro/) [40].

### RT-PCR Analysis of NcSP75, 70, and 38 Transcripts in Different Organs

Total RNA was extracted from salivary glands, stomach, Malpighian tubules, and fat bodies using RNeasy Mini Kit (Qiagen, Valencia, CA, USA) following manufacturer’s protocol. RNA was reverse-transcribed using oligo-dT primers. Each cDNA sample was amplified using Quick Taq HS DyeMix (TOYOBO, Osaka, Japan) with specific primers. Primer pairs for *NcSP75*, *NcSP70*, *NcSP38*, and a reference gene that codes the ribosomal protein L19 [32], are shown in S1 Table. PCR was performed for 25 cycles (*NcSP75*, and *NcSP70*, and *RpL19*) or 30 cycles (*NcSP38*) as follows: 30 s at 94°C, 30 s at 55°C and 40 s at 68°C. Amplified DNA was analyzed on a 1.2% agarose gel.

### Multiple Sequence Alignments and Phylogenetic Analysis of NcSP38

Phylogenetic analysis was performed using Molecular Evolutionary Genetics Analysis 6 (MEGA 6) [41]. Sequences were aligned using Clustal Win MEGA. After manual adjustments, conserved regions of glycoside hydrolase family 5 (GH5) sequences, in which the assignment of positional homology was possible, were used for tree construction, while all other regions were excluded. A tree was constructed using the neighbor-joining method with a Poisson correction model. Gaps and missing data were eliminated and statistical analysis was performed with at least 1000 bootstrap repetitions. Insect cellulase cDNA sequences were used for phylogenetic analysis: *N*. *cincticeps*, *NcSP38* (LC009514), *N*. *cincticeps* EGase (TsukubaH.comp12770), *H*. *coagulate* (EG371534), *Apriona japonica* (AH15747), *Apriona germari* (AAX18655.1), *Anoplophora chinensis* (AFN89566.1), *Oncideres albomarginata chamela* (ADI24131), *Psacothea hilaris* (BAB86867), *Hypothenemus hampei* (ACU52526.1), *Streptomyces sviceus* (WP_007385903.1), *Streptomyces violaceusniger* (WP_014179470), and *Paenibacillus polymyxa* (WP_019686064.1).

## Results

### Major Proteinaceous Components of Watery Saliva Separated by SDS-PAGE

**Fig 1:**
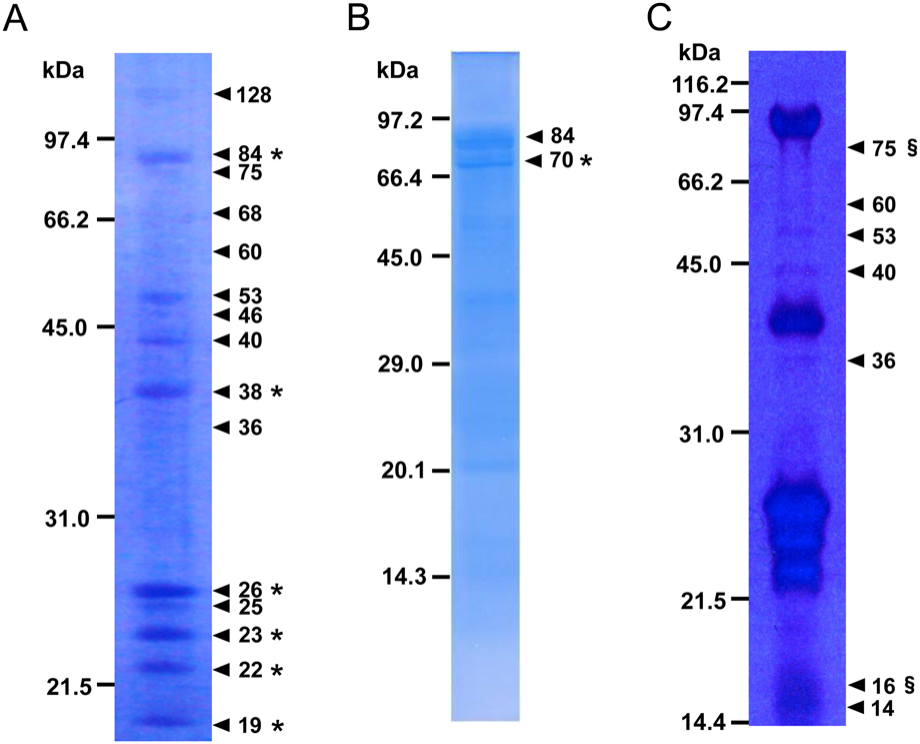
Proteins in watery saliva collected from 5% sucrose diet that *N*. *cincticeps* had fed on. **(A)** Salivary proteins electroblotted onto a PVDF membrane after being subjected to SDS-PAGE (12.5% polyacrylamide gel) under reducing conditions and **(B)** SDS-PAGE (15% gel) under non-reducing/non-heating conditions. N-terminal sequences of protein bands marked with asterisks were determined using a gas-phase protein sequencer. **(C)** Salivary proteins separated on a gel after being subjected to SDS-PAGE (13% gel) under reducing conditions. Protein bands marked with section signs were identified using MS-MS analysis combined with our EST database.

N-terminal amino acids of salivary proteins separated by SDS-PAGE under reducing or non-reducing conditions were sequenced using a protein sequencer. Fifteen protein bands at molecular masses of 128, 84, 75, 68, 60, 53, 46, 40, 38, 36, 26, 25, 23, 22, and 19 kDa were visible under reducing conditions (Fig 1A). Two proteins with 84 and 70 kDa were visible under non-reducing conditions (Fig 1B). N-terminal sequence was determined from seven major *N*. *cincticeps* salivary proteins, NcSP84, NcSP70, NcSP38, NcSP26, NcSP23, NcSP22, and NcSP19 (asterisks in Fig 1A and 1B). The remaining proteins, which could be N-terminal blocked, were excised from SDS-PAGE gel and analyzed by MS/MS. Analysis identified NcSP75 and NcSP16, which were matched to salivary gland ESTs of *N*. *cincticeps* (section signs in Fig 1C).

### N-terminal Sequences, Tandem MS and cDNA

Table 1 shows the peptide sequences obtained by protein sequencer and tandem MS. Seven out of nine proteins corresponded to EST sequences exclusively derived from the salivary gland of *N*. *cincticeps*. NcSP70 was matched against ESTs from the midgut, the ovary and salivary glands. NcSP38 showed no match against any EST. Nearly full-length cDNAs of these salivary proteins were obtained by 5′ and 3′ RACE-PCR based on assembled EST contigs or sequence determined by degenerate PCR (S1 Table).

**Table 1:** Identification of major saliva proteins of *N*. *cincticeps* using SDS-PAGE combined with protein sequencer and MS.

M_r_ (kDa)	N-terminal sequence ^a^	Peptide sequences	MASCOT total ion score/ expect	Protein homologous to translated nucleotide sequence	E-value	MW /pI(deduced mature protein)	No. of ESTs ^b^	NGlyc	OGlyc
84	SSDTVPAEVQTIVKT			calcium-binding protein (AB618633.1) (Hattori et al., 2012)	0.000	77544.9 / 4.7	876 SG	0	0
75	not determined	K.MSEQEMESK.I K.GVINVLLTAAGK.G K.GGGITDKMSEQEMESK.I	51 / 1.6	liver stage antigen 3 [*Plasmodium falciparum*]	8e-05	78253.9 / 4.8	73 SG	2	0
70	GPAPKTLKL			transferrin [Rhodnius prolixus]	0.00	71886.8 / 6.0	3 SG, 2 MG, 2 OV	3	0
38	ANLPGGKQNFPRLR			cellulose-binding protein [*Streptomyces* sp.]	3e-118	37865.6 / 5.3	0	1	0
26	FSQXNTETLNKIKAS			unknown		20198.0 / 8.2	70 SG	0	0
23	TEVQDVSTIXNILLL			unknown		19019.4 / 5.0	16 SG	0	0
22	AHPQVEHVDXVPQNV			unknown		18867.8 / 6.0	93 SG	0	0
19	AXASTEVKASDASAY			unknown		11069.2 / 9.0	93 SG	0	9
16	not determined	K.HFCAVHYGAK.C K.DFNAEQDGKVFEVK.T K.CVTYTNEVDTHNQAK.A K.GNNAIAWMSVDNCSGLK.D K.VLFTTTPHFLANIQGNPVADTK.H	73 / 0.4	unknown		15010.9 / 6.6	105 SG	0	0

^a^ Protein band numbers correspond to those described in Fig 1.

^b^ Total of 25061 ESTs of *N*. *cincticeps* for salivary gland (8436), midgut (5908), micetome (3095), ovary (4364) and testis (3258) of *N*. *cincticeps*. NcSp84, 75, 26, 23, 22, 19, and 16 were matched to ESTs which are exclusively derived from salivary gland. Only NcSP70 matched to ESTs which are derived from various organs including salivary gland (SG), midgut (MG) and ovary (OV).

All major putative proteins constructed had a signal peptide indicative of secretion, and contain no hydrophobic transmembrane segments (Table 1), suggesting that the entire protein is transferred into the lumen of the endoplasmic reticulum (ER) after synthesis. NetNGlyc 1.0 software identified 2 potential N-glycosilation sites in NcSP75 and 1 in NcSP38, while NetOGlyc 3.0 identified nine potential O-glycosylation sites in NcSP19. NcSP84, reported as a Ca^2+^-binding protein [32], was the most predominant protein. NcSP75 was homologous to liver stage antigen 3 in *Plasmodium falciparum* (E-value: 8e-05), and NcSP70 had a strong sequence homology with transferrin of *Rhodnius prolixus*. NcSP38 was highly homologous to cellulose-binding protein NRRL F-5123 of *Streptomyces* sp. (E-value: 3e-118) and endo-1, 4-beta-glucanase of *Streptomyces bingchenggensis* (E-value: 2e-117), which belongs to GH5 (IPR001547, pfam00150). Other major proteins showed weak or no homology to any previously reported sequences. TBLASTn search against NCBI-EST database showed that NcSP38 (E-value: 2e-04), NcSP23 (E-value: 4e-09, and NcSP19 (E-value: 2e-06) had high similarity to cDNA from salivary glands of glassy-winged sharpshooter, *Homalodisca vitripennis* (formerly *H*. *coagulata*), a member of Auchenorrhycha suborder, in which *N*. *cincticeps* belongs to also.

The nucleotide sequence data of *NcSP75*, *NcSP70*, *NcSP38*, *NcSP26*, *NcSP23*, *NcSP22*, *NcSP19*, and *NcSP16* have been submitted to DNA databank of Japan (DDBJ) under accession no. LC009512–LC009519, respectively.

### Expression of NcSP70 and 38 Transcripts in Different Tissues

**Fig 2:**
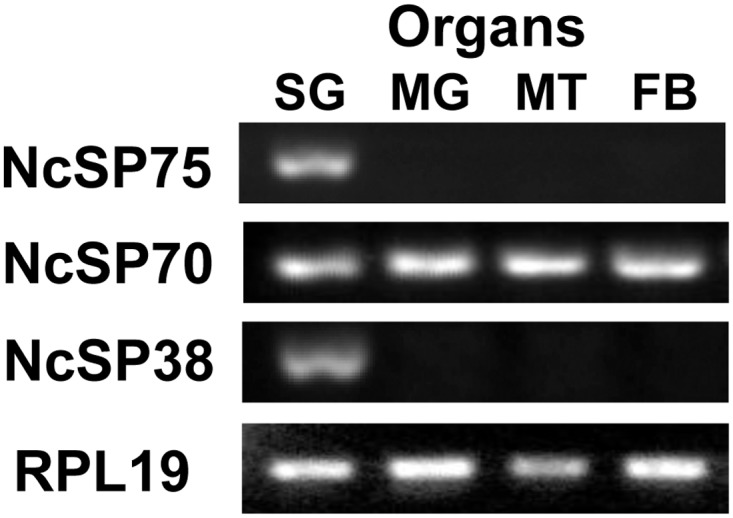
Expression of *NcSP75*, *NcSP70*, *NcSP38* genes in different organs. RT-PCR on total RNA isolated from salivary glands (SG), midgut (MG), Malpighian tubules (MT), and fat bodies (FB) of adult females. Each gene was amplified with gene-specific primers. Ribosomal protein L19 primers were used as control in equal template amounts.

Transcripts of almost all major salivary proteins matched against many ESTs derived exclusively from the salivary gland (Table 1). However, NcSP70 (transferrin) transcript matched against 8 ESTs in total, 2 from the midgut and 3 from the ovary and 3 from the salivary gland. NcSP38 mRNA sequence did not match against any EST in the database. Expression analysis in different tissues was performed for NcSP70 and NcSP38 transcripts using RT-PCR. NcSP70 was universally expressed in salivary glands, midgut, Malpighian tubules, and fat bodies examined, while NcSP38 was specifically expressed in the salivary glands (Fig 2).

### Multiple Sequence Alignments and Phylogenetic Analysis of NcSP38 (GH5)

**Fig 3:**
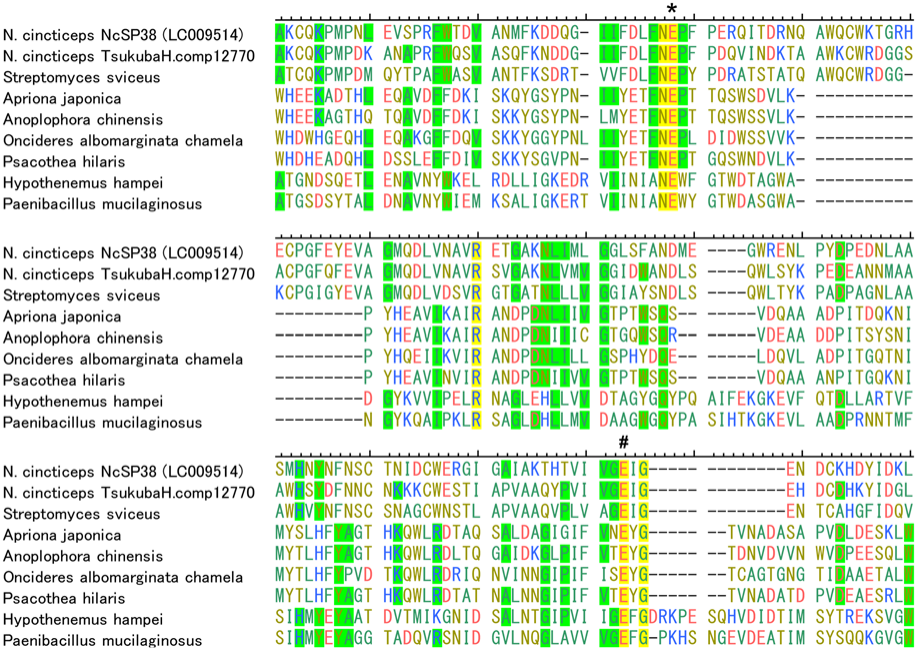
Multiple alignment of GH5 family protein sequences identified from *Nephotettix cincticeps* with GH5 proteins of five coleopteran insects and two bacterial GH5 proteins. Multiple alignments was performed using deposited data from Hemiptera: *Nephotettix cincticeps* (NcSP38:LC009514, and TsukubaH.comp12770), Coleoptera: *Hypothenemus hampei* (ACU52526), *Apriona japonica* (AHI15747), *Anoplophora chinensis* (AFN89566), *Psacothea hilaris* (BAB86867), *Oncideres albomarginata chamela* (ADI24131), Bacteria: *Paenibacillus mucilaginosus* (WP_013917961), *Streptomyce rimosus* (WP_030596221). Asterisk (*) and number sign (#) on the amino acid residues represent the catalytic nucleophile and catalytic proton donor, respectively (based on Eyun et [58]).

**Fig 4:**
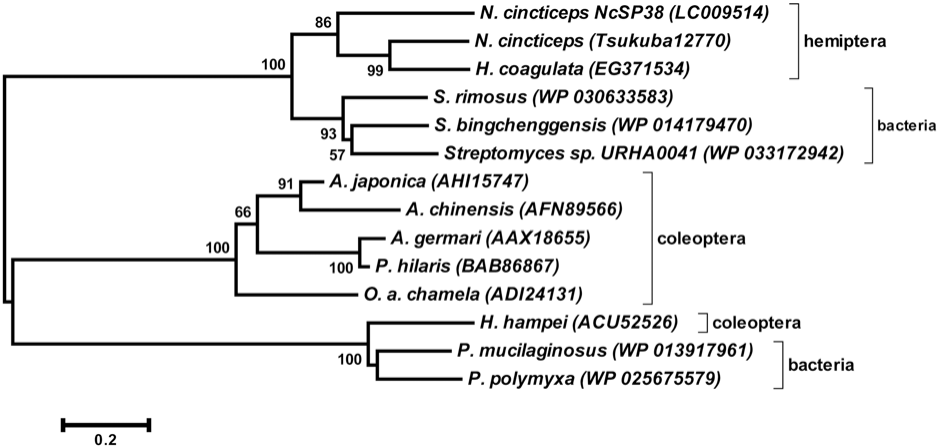
The phylogenetic tree of GH5 family protein sequences identified from *Nephotettix cincticeps* with GH5 proteins of six coleopteran insects and five bacterial GH5 proteins. Phylogenetic analysis was performed using deposited data from Hemiptera: *Nephotettix cincticeps* (NcSP38:LC009514, and TsukubaH.comp12770), *Homalodisca coagulata* (EG371534), Coleoptera: *Hypothenemus hampei* (ACU52526), *Apriona germari* (AAX18655), *A*. *japonica* (AHI15747), *Anoplophora chinensis* (AFN89566), *Psacothea hilaris* (BAB86867), *Oncideres albomarginata chamela* (ADI24131), Bacteria: *Paenibacillus mucilaginosus* (WP_013917961), *P*. *polymyxa* (WP_025675579), *Streptomyce rimosus* (WP_030596221), *S*. *bingchenggensis* (WP_01417940), and *Streptomyces sp*. URHA0041 (WP_033172942). These sequences were aligned using default condition of MEGA6 [41]. All positions containing gaps and missing data were eliminated. Neigbor-joining (NJ) analysis was conducted based on 164 amino acid sequences using MEGA6. Bootstrap values were calculated for 1,000 replications.

As described above, NcSP38 showed significant similarities to endo-1,4-beta-glucanase which belongs to GH5, and was also homologous to cDNA from the salivary glands of glassy-winged sharpshooter, *Homalodisca vitripennis* (EG371534 and DN198573.1). GH5 are cellulases found in microbes and some coleopteran insects, while insect cellulases usually belong to GH9 [42]. Therefore, multiple sequence alignments and phylogenetic analysis of GH5 gene was performed. Sequence similarity and phylogenetic analysis revealed that NcSP38 and its homologous genes in *H*. *vitripennis* were more related to GH5 of *Streptomyces*, rather than the GH5 of coleopteran insects (Figs 3 and 4).

### Identification of Salivary Proteins Using Gel-free Based Nano LCMS/MS Analysis

Gel-free proteome analysis was performed to cover the broad range of salivary proteins, as RNA-sequencing data of *N*. *cincticep*s salivary gland is currently available. Comparison of MS/MS data against RNAseq database for *N*. *cincticeps* using an internal Mascot search routine yielded the identification of 62 additional protein species. About 60% of the salivary proteins showed a significant match to database enzymes or other bioactive proteins including lipase, carbohydrate kinase, heat shock protein 70, chromosome segregation protein smc2 (ATPase), carbonic anhydrases, aminopeptidase, and regucalcin (Table 2 and S3 Table). The remaining salivary proteins had no homology to any previously reported sequences (S2 Table and S3 Table). TBLASTn search against NCBI-EST database revealed that in addition to NcSP38, NcSP23, and NcSP19, nineteen salivary components that were identified by gel free analysis had similarities to the translated amino acid sequences from salivary gland cDNA of *H*. *vitripennis*

**Table 2:** Proteins identified in watery saliva of *N*. *cincticeps* by gel-free based nano LC-MS/MS.

Contig-ID	Protein Identification	NCBI accession no.	total ion score	No. of unique peptide matches ^a^	MW	pI	No. of ESTs ^b^	SG-EST ^c^(*H*.*vtripenis*)	Secretion Signal	InterPro ID
TsukubaH.comp12770_c0_seq1	endo-1,4-beta-glucanase (GH5 cellulase)	gi|503945476	3343	13	41608.7	5.4	0	YES	YES	IPR001547
TsukubaH.comp11865_c0_seq1	deoxyribonuclease I	gi| 5881881	957	21	49288.1	7.4	1 SG	YES	YES	IPR001604 IPR020821
TsukubaH.comp10744_c0_seq1	venom protein M precursor	gi| 238908536	417	10	20988.3	7.6	1 SG, 1 MY	NO	YES	NO
TsukubaH.comp13567_c0_seq1	Laccase-4	gi|646702640	423	11	80144.3	7.1	4 SG	YES	YES	IPR011707 IPR001117 IPR011706
TsukubaH.comp14402_c0_seq1	hypothetical protein BRAFLDRAFT_89329	gi|260826744	231	5	31002.1	6.1	1 SG, 1 MY	YES	YES	NO
TsukubaH.comp12976_c0_seq1	neuroendocrine convertase 1-like	gi|383857707	182	5	74497.1	6.5	0	YES	YES	IPR009020 IPR000209 IPR002884
TsukubaH.comp7411_c0_seq1	lysozyme 3-like	gi|345498458	172	3	14853.9	4.5	4 SG	NO	YES	IPR008597
TsukubaH.comp4366_c0_seq1	malate dehydrogenase	gi| 156553655	154	6	>36019.6	~8.9	2 SG, 1 MY	NO	YES	IPR001236 IPR015955
TsukubaH.comp11079_c0_seq1	heat shock protein 70	gi|292606983	145	7	71293.7	5.4	3 MG, 11 OV	NO	NO	IPR029047 IPR029048
TsukubaH.comp13568_c0_seq1	Laccase-1S	gi|295292757	135	7	77374.1	7.8	2 SG	NO	YES	IPR008972 IPR011707 IPR001117 IPR011706
TsukubaH.comp11772_c0_seq1	regucalcin-like	gi| 572263243	133	6	35463.0	5.4	40 SG	NO	YES	IPR011042 IPR013658
TsukubaH.comp12022_c0_seq1	hydroxyacylglutathione hydrolase,	gi| 498944321	131	9	34043.0	6.6	2 SG, 1 MG,1 OV	NO	NO	IPR017782 IPR001279
TsukubaH.comp3954_c0_seq2	carbonic anhydrase 7-like	gi| 328702174	123	5	31363.6	6.2	9 SG	YES	YES	IPR001148
TsukubaH.comp12518_c0_seq1	hypothetical protein DICPUDRAFT_150209	gi| 330796426	119	4	>76970.6	~4.9	1 SG	YES	NO?	NO
TsukubaH.comp10065_c0_seq1	apolipoprotein D-like	gi|662205419|	102	4	30993.0	5.3	1 SG	NO	YES	IPR011038 IPR012674
TsukubaH.comp13792_c0_seq1	glyceraldehyde-3-phosphate dehydrogenase	gi|53830712	102	7	>35642.8	~8.3	1 SG, 3 MG,1 OV	NO	NO	IPR020828 IPR020829
TsukubaH.comp10607_c0_seq1	pectin lyase	gi|660962455	92	4	41124.1	7.7	0	NO	YES	IPR011050 IPR002022
TsukubaH.comp13482_c0_seq1	aminopeptidase N-like	gi|328718942	89	9	105968.0	5.4	1 SG	NO	YES	IPR014782 IPR024571
TsukubaH.comp11730_c0_seq1	coiled stalk of trimeric autotransporter adhesin family protein	gi|686968069	87	2	60865.1	9.3	6 SG	YES	YES	NO
TsukubaH.comp12456_c0_seq1	pyruvate dehydrogenase E1	gi|332020438	83	4	>43158.1	~8.4	0	NO	NO	IPR029061 IPR001017
TsukubaH.comp13746_c0_seq1	nucleoside diphosphate kinase	gi|90819960	80	8	17141.7	8.9	6 SG, 6 MG,8 OV, 2 MY	NO	YES	IPR023005
TsukubaH.comp12767_c0_seq1	collagen alpha-1 chain-like isoform X6	gi|548334138	76	3	22783.5	8.7	2 OV	NO	YES	IPR008160
TsukubaH.comp3925_c0_seq2	carbonic anhydrase VII	gi| 158254178	68	6	>32852.3	~5.9	13 SG	YES	NO	IPR001148
TsukubaH.comp4557_c0_seq1	triosephosphate isomerase	gi|565321260	67	3	18214.8	5.5	3 MG, 5 OV, 3 MY, 1 TE	NO	NO	IPR013785
TsukubaH.comp13820_c0_seq1	hypothetical protein KGM_09817	gi| 357622196	65	6	29718.2	7.0	2 SG	YES	YES	NO
TsukubaH.comp12438_c0_seq2	Ubiquitin	gi|56199552	61	2	>13450.6	~9.3	1 SG	NO	NO	IPR029071 IPR000626
TsukubaH.comp14120_c0_seq1	Unknown (Apolipophorin-III)	gi|146285338	59	2	22260.4	5.8	2 SG, 1 MG,1 OV, 2 MY	NO	YES	G3DSA: 1.20.120.20
TsukubaH.comp13035_c0_seq1	enolase	gi|562745090	58	7	46783.4	6.0	2 SG, 8 MG,21 OV, 4 TE	NO	NO	IPR029017 IPR029065
TsukubaH.comp11046_c0_seq1	pancreatic lipase	gi| 328710007	42	3	36384.9	5.6	11 SG, 4MG	NO	YES	IPR029058 IPR013818
TsukubaH.comp13308_c1_seq7	superoxide dismutase	gi|149898934	50	3	15723.6	6.2	2SG, 2OV	NO	NO	IPR001424
TsukubaH.comp3848_c0_seq1	phosphatidylethanolamine-binding protein	gi|157133196	45	3	25214.6	7.7	2 OV, 7MY	NO	YES	IPR008914
TsukubaH.comp13467_c0_seq1	salivary inositol polyphosphate 5-phosphatase	gi|149689206	33	3	32595.2	6.8	43 SG	NO	YES	IPR005135 IPR000300
TsukubaH.comp11983_c0_seq7	NADP-dependent malic enzyme	gi|646663160	32	5	73858.4	6.3	1 OV, 1 TE	NO	NO	IPR012301 IPR016040
TsukubaH.comp10419_c0_seq1	hypothetical protein TcasGA2_TC007567	gi|270005504	30	3	37340.0	9.3	2 SG	NO	NO	IPR029058
TsukubaH.comp3952_c0_seq1	hypothetical protein UCREL1_21	gi|629638332	28	2	22813.9	5.6	32 SG	NO	YES	NO
TsukubaH.comp10152_c0_seq1	arginine kinase	gi|540361100	26	5	40134.6	6.0	14 SG, 39 MG, 24 OV, 12 MY	NO	NO	IPR022413 IPR014746
TsukubaH.comp7644_c0_seq1	hypothetical protein PFL1_03272	gi|630965144	23	3	23904.0	8.9	1 SG	NO	YES	NO
TsukubaH.comp3946_c0_seq1	fructose 1,6-bisphosphate aldolase	gi|46561746	19	2	39665.2	7.1	3 OV, 6 MY	NO	NO	IPR013785
TsukubaH.comp13836_c0_seq1	ribosomal protein L12	gi|24200936	18	3	17945.0	9.2	0	NO	NO	IPR020784 IPR020783
TsukubaH.comp4079_c0_seq1	actin-depolymerizing factor 1	gi|506965820	16	4	17054.3	6.7	5 MG,14 OV, 3 MY	NO	NO	IPR029006 IPR002108

Proteins, which matched to peptides from salivary gland RNAseq data of *N*. *cincticeps*, were searched against the NCBI non-redundant database.

^a^ The unique matched peptides are shown in S3 Table.

^b^ The number of matched ESTs of *N*. *cincticeps*, which are derived from various organs such as the salivary gland (SG), midgut (MG), ovary (OV), mycetome (MY), and testis (TE).

^c^ YES: matched to ESTs from the salivary gland of *H*. *vtripenis*.

### Predictive Molecular Weight and pI of Salivary Proteins in Watery Saliva

Putative mature proteins identified from *N*. *cincticeps* saliva had predictive molecular weight ranging from 1.8 to 106 kD and isoelectric point (pI) values ranging from 4.1 to 9.3 (Table 2 and S1 Fig). Putative proteins with 10–20 kDa or 5–7 of pI accounted for a relatively high percentage of all sequences that covered open reading frames (ORFs). Aphid saliva also contains proteins of a broad molecular weight range [15, 16, 18, 21, 19–,21]. In *M*. *persicae*, a saliva protein with a mass of 3–10 kDa is known to induce defense responses in *Arabidopsis thaliana* [7].

## Discussion

To date, a limited number of studies have been conducted on the salivary components of phytophagous Auchenorrhyncha species [43–45]. Comparison of salivary components between Auchenorrhyncha, including leafhoppers, with those of Sternorrhyncha, including aphids, is necessary to comprehensively understand the basal functions of salivary components in feeding from vascular bundles, especially from phloem.

In an attempt to identify the watery saliva components of *N*. *cincticeps*, proteins recovered from a 5% sucrose diet solution was subjected to SDS-PAGE. N-terminus of separated protein was sequenced by Edman degradation, while N-terminal blocked proteins were analyzed using LC-MS/MS on tryptic peptides from salivary proteins in-gel samples. Out of the 16 proteins that were visualized with CBB, 9 that included a previously reported protein with a molecular mass of 84 kDa were identified as major components. A protein of 84 kDa (NcSP84, AY087460) was a unique Ca^2+^-binding protein with multiple EF hand motifs [32]. This protein was injected into sieve tubes and might inhibit local occlusion responses in the sieve tubes of rice plant [32]. Some Ca^2+^-binding proteins that counteract sieve-plate occlusion have been detected in the watery saliva of *M*. *viciae* [46], while regucalcin and calreticulin were identified in the saliva of *Acyrthosiphon pisum* [16] and *Diuraphis noxia* [19], respectively. Gel-free analysis, unexpectedly, indicated that regucalcin was also a component in *N*. *cincticeps* saliva. This protein does not contain EF-hand motif of Ca^2+^-binding domain [47]. Two kinds of Ca^2+^-binding proteins found in this species could inhibit different signal cascades that are mediated by Ca^2+^ in different process of feeding. Similarly, in addition to laccase-1S (BAJ06131, TsukubaH.comp13568) [29, 30], which has been identified before in the saliva of *N*. *cincticeps*, a new laccase (TsukubaH.comp13567) was also identified by gel-free analysis (Table 2). Different laccase proteins may exhibit different substrate specificitiy. Salivary laccase probably has two functions, solidification of sheath saliva and rapid oxidation of plant phenolics [29].

NcSP70 was matched against transferrin, which has not been previously identified in any insect saliva. Unlike the other major components of *N*. *cincticeps* saliva, transcripts of this protein were universally distributed in different tissues (Table 2, Fig 2). Transferrin has been characterized in many insect species, including *Protaetia brevitarsis* [48], *Riptortus clavatus* [49], and *Bombyx mori* [50]. Insect transferrin is known as an iron transporter, an antibiotic agent, a juvenile hormone-regulated protein [48, 49, 51]. Additionally, this protein was shown to have an antioxidant function, and inhibit stress-induced apoptosis by diminishing the Fenton reaction via the binding of iron in *Protaetia brevitarsis* [52]. In general, these functions are considered to work in various tissues of the insect body. So far, transferrin has not been identified from insect saliva. Salivary transferrin of *N*. *cincticeps* may bind free Fe (II) in the plant cell, and eventually suppress the generation of reactive hydroxyl radical in broken cells by Fenton's reaction. Indeed, ferrous ion is present in rice phloem sap [53].

*NcSP38* and TsukubaH.comp12770 were matched against sequences annotated as GH5 cellulase. Additionally, *NcSP38* mRNAs showed homology to cDNAs from the salivary glands of glassy-winged sharpshooter, *H*. *vitripennis*. So far, cellulase genes encoding GH5 have been reported exclusively for coleopteran insects, while GH9 cellulases are distributed in insect orders as Orthoptera, Blattaria, Phthiraptera, Hemiptera, Coleoptera, and Hymenoptera, but do not have any sequence similarities with GH5 [42, 54–58]. The present study showed that another hemipteran insect also has GH5 in the saliva or salivary glands. Unexpectedly, hemipteran GH5 sequence is phylogenetically closer to streptomyces (actinomycetes) GH5 sequences than coleopteran insect GH5. The question that arises is if hemipteran GH5 found in salivary glands comes from symbiotic microorganisms. According to Noda et al. [59], however, *N*. *cincticeps* harbors two symbionts, *Sulcia* and *Nasuia*, in the bacteriome and another symbiont, *Rickettsia*, systemically, but not any microorganisms have been detected in salivary glands [59]. RT-PCR revealed that *NcSP38* mRNA is expressed exclusively in the salivary glands (Fig 2), while gene amplification was equally frequent in salivary gland, stomach, Malpighian tubules, and fat bodies, as shown by genomic DNA-PCR (data not shown). Our results may indicate that this enzyme is not derived from symbiotic bacteria inhabiting in the salivary glands. It is possible that this gene may not be inherited vertically from a common ancestor of hemipteran and coleopteran species, but horizontally from actinomycetes to the common ancestor of leafhoppers and sharpshooters. Indeed, GH5 family gene identified in coffee berry borer, *H*. *hampei* was suggested to be of bacterial origin [60]. However, further research is required to examine the possibility of a horizontal transfer of *NcSP38* gene, as well.

Backus et al. [45] reported that cellulase (endo-β-1, 4-glucanase: EGase) activity was detected in salivary sheaths of *H*. *vitripennis*, and reported to act on β-1,4-glucosidic linkage of hemicellose xyloglucan polysaccharide of plant cell walls, in combination with β-glucosidase. Therefore, cellulase of *N*. *cincticeps* may participate to the lysis of cell walls in the phloem and xylem. Interestingly, a large quantity of xyloglucans was recently identified in the phloem cell walls of Poaceae plant species, including the rice plant [61, 62].

NcSP19 was predicted to have nine mucin-type GalNAc (N- acetylgalactosamine) O-glycosylation sites, and may be associated with salivary sheath material, in which a neutral mucosubstance was detected [2].

Sixty-three additional proteins were identified by gel-free proteome analysis of *N*. *cincticeps* saliva. Venom protein M precursor (TsukubaH.comp10744), shown in Table 2, is a cysteine-rich protein that contains approximately 17% cysteine residues in its mature sequences. A protein with a high content of cysteine has been identified in the saliva of *A*. *pisum* [16], although it is not homologous to the venom protein. The conversion of sulfhydryl groups to disulfide linkages in cysteine residues of proteins is assumed to lead to the formation of a solid sheath [63, 64]. Two carbonic anhydrases, aminopeptidase N-like, apolipoprotein, and regucalcin have been commonly found in the saliva of *N*. *cincticeps* and aphids [15, 16, 19–21]. It is assumed that carbonic anhydrase maintains the pH of the phloem during feeding, and also suppress salicylic acid (SA) response [20]. Aminopeptidase was suggested to catalyze the hydrolysis of toxic proteins, as plant lectins [65]. Apolipoproteins, lipid transport proteins, may capture plant defensive molecules and counteract lipid-based plant defense [20]. *N*. *cincticeps* saliva did not contain glucose dehydrogenase, which is a major constituent of aphid saliva [15, 16, 18–21]. Glucose dehydrogenase has been known to reduce host-plant produced quinone and phenoxy radicals, and thereby protect from host defenses [20]. On the contrary, laccases (diphenol oxidases) found in *N*. *cincticeps* saliva have not been identified in aphid saliva by proteome analysis, although their existence has been suggested by enzymatic methods [12].

Out of 71 proteins identified in *N*. *cincticeps* saliva, 22 proteins, including NcSP38, 23, and 19 showed similarity to salivary gland cDNAs of *H*. *vitripennis* (Table 1, Table 2 and S2 Table). Both insect species belong to vascular bundle-feeding Auchenorrhynchans, although *H*. *vitripennis* ingests xylem sap almost exclusively [66, 67]. Therefore, if *H*. *vitripennis* discharges these homologs in the saliva as *N*. *cincticeps* does, proteins found in the saliva of both species, such as deoxyribonuclease (endonuclease), laccase and carbonic anhydrases, may serve in feeding on xylem rather than phloem, and/or from penetrating plant tissues, prior to vascular bundle feeding. On the contrary, aminopeptidase and regucalcin, found in aphids did not match against ESTs of *H*. *vitripennis*, probably because these proteins may be associated with stable feeding on phloem.

Kalume et al. [36] reported that gel-free and in-gel approaches are complementary for the identification of salivary gland proteins, because the use of one strategy alone could lead to loss of some proteins identified by the other one. In our study, 7 out of 9 proteins which were identified by the in-gel method were also identified by the gel-free method, but the other proteins (NcSP84 and NcSP75) were identified only by the in-gel method (S4 Table).

The salivary proteins were collected from the sucrose solution that leafhoppers had fed upon, so they were expected to be different than those from the saliva composition ejecting during feeding from plants. Moreno et al. [7] reported that watery saliva, along with gelling saliva, is secreted by aphids from the onset of stylet penetration into the plant tissue. If this were also true for leafhoppers, some components of watery saliva would have been incorporated into salivary sheaths and not recovered from the fed-diet. Furthermore, it should be noted that relatively low-concentrated or dissolved proteins were not fully recovered from the diet solution probably because they were either adsorbed onto the membrane and ultrafiltration apparatus or they were digested during the collection and concentration process. For example, cytochrome P450 has been identified in the secreted saliva of aphids such as *Acyrthosiphon pisum*, *Myzus persicae*, *Sitobion avenae* [19, 21, 65], or *Diuraphis noxia* [19]. This protein is probably missing in our proteome analysis of *N*. *cincticeps* saliva, because their transcripts were found to be relatively highly expressed in the salivary glands of this insect according to RNA-seq results [33]. Therefore, complementary approaches using transcriptome sequencing and proteomics of samples from salivary glands are essential for obtaining the full profile of salivary components.

Overall, our study showed that total of 71 proteins identified in *N*. *cincticeps* saliva contain functional proteins in common with those in the saliva of aphids, typical phloem feeders, and those correspond to salivary gland EST sequence of *H*. *vitripennis*, a xylem feeder. Twenty-eight unknown proteins, including major proteins, appeared to be unique and species-specific. Effective knockdown of novel salivary proteins in combination with the examination of feeding response are required to elucidate the precise role of individual salivary components in leafhopper feeding. Moreover, it will provide an insight into how *N*. *cincticeps* counteract defensive responses of rice and other Poaceae host plants, thus accomplishing successful and sustained ingestion of phloem. This data will help in developing tools to control this insect and other vascular bundle-feeding insects in the future.

## Supporting Information

S1 Fig Predictive molecular weight and isoelectric point (pl) values of salivary proteins in watery saliva. Molecular weight and pI were determined for mature proteins for which a cDNA sequence covering ORF is available (N = 55).
(TIF)

S1 Table Primer sequences used in this study. (DOCX)

S2 Table Unknown proteins identified in watery saliva of *N*. *cincticeps* by gel-free based nano LC-MSMS. Unknown proteins matched to peptides from salivary gland RNAseq data of *N*. *cincticeps*. ^a^ The matched unique peptides are shown in S3 Table. ^b^ The number of matched ESTs of *N*. *cincticeps*, which are derived from the salivary gland (SG). ^c^ YES: matched to ESTs from the salivary gland of *H*. *vtripenis*.
(DOCX)

S3 Table Matched unique peptides identified in the saliva proteins of *N*. *cincticeps* by gel-free based nano LC-MSMS. (DOCX)

S4 Table Proteins identified in watery saliva of *N*. *cincticeps* by both in-gel and gel-free methods. NcSP84 (AB618633) and NcSP75 (LC009515) were identified only by in-gel method (see Table 1). ^a^ The unique matched peptides are shown in S3 Table. ^b^ YES: matched to ESTs from the salivary gland of *H*. *vtripenis*.
(DOCX)
