# Supplementary figures and images for: Proteome Analysis of Watery Saliva Secreted by Green Rice Leafhopper, Nephotettix cincticeps
Source: PLoS One. 2015 Apr 24;10(4):e0123671. doi: 10.1371/journal.pone.0123671 (PMC4409333; doi:10.1371/journal.pone.0123671)

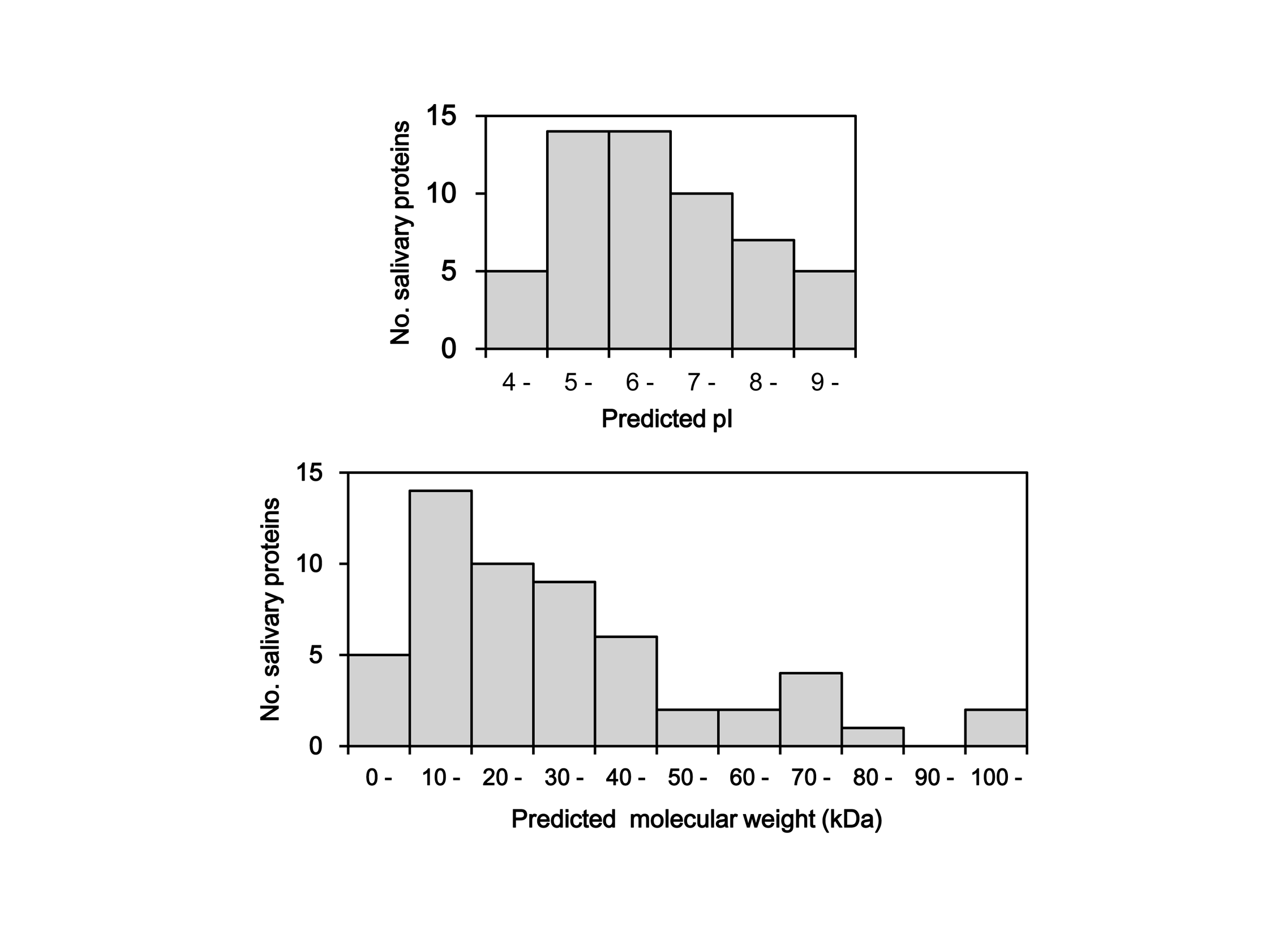

Supplement: S1 Fig — Molecular weight and pI were determined for mature proteins for which a cDNA sequence covering ORF is available (N = 55). (TIF) [file pone.0123671.s001.tif]
