# Supplementary material for: Proteome Analysis of Watery Saliva Secreted by Green Rice Leafhopper, Nephotettix cincticeps
Source: PLoS One. 2015 Apr 24;10(4):e0123671. doi: 10.1371/journal.pone.0123671 (PMC4409333; doi:10.1371/journal.pone.0123671)
Supplement: S1 Table — (DOCX) [file pone.0123671.s002.docx]

**S1Table. Primer sequences for *N. cincticeps* major saliva proteins.**

| Name | Orientation | Sequences (5′ to 3′) |
| --- | --- | --- |
| 3′- and 5′-RACE | | |
| NcSP16-R | Reverse | GTGCCGATGGTCCGAGGAAGTAGAC |
| NcSP19-R | Reverse | AGGCAGGAGCCAACAGAGCGATG |
| NcSP22-F | Forward | CGAAAATGCATACCATTACTTCTG |
| NcSP22-R | Reverse | CATATATTTAAACTAATTCATTGAC |
| NcSP23-F | Forward | GATGGCCCGGATCATCGTTACATTC |
| NcSP23-F-nest | Forward | AGATGATGCAAAATACGAATGG |
| NcSP23-R | Reverse | CGGGCCATCAATTCCTTGTTCTGTT |
| NcSP23-R-nest | Reverse | GCTGGTAGTCGGGGAGATGATTA |
| NcSP26-F | Forward | CGTGACTTGTACTGACAAAAAG |
| NcSP26-R | Reverse | TTGACGCCCACTGGGAAGTAGAAGG |
| NcSP38-F | Forward | CCIGGIGGIAARCARAAYTTYCCIMGIYT |
| NcSP38-R | Reverse | CGTTTATCGCCTGCTTGTAATTGTC |
| NcSP75-F | Forward | TGCCAGCAAGAGTGTTGCAGAGAACTTA |
| NcSP75-F-nest | Forward | TAGGTCTGATGAAGTAGGCACAG |
| NcSP75-R | Reverse | TCTTCTTCTTCATGGACTCCGCTTCCTC |
| NcSP75-R-nest | Reverse | AGGTGGCTTGGAGACGAACTGAAC |
| Full-length cDNA |  |  |
| f-NcSP16F | Forward | GACTGAGACTCACAATCATGTTC |
| f-NcSP16R | Reverse | ACAGAAACTCTACTTCTCTGTATCC |
| f-NcSP19F | Forward | ACATCAGTCAGTCTGCTCCCAGCAT |
| f-NcSP19R | Reverse | TTCCAGTCAGTTCACAGACATTTAC |
| NcSP22-fFR | Forward | CGAAAATGCATACCATTACTTCTG |
| NcSP22-fRV | Reverse | CATATATTTAAACTAATTCATTGAC |
| f-NcSP23F | Forward | AACAGCCTTTGAACAGCTGATCC |
| f-NcSP23R | Reverse | CCAAAGTATAATGCAGCGTTTATT |
| f-NcSP26F | Forward | AGTCAACAACTCTTCAGAGACTC |
| f-NcSP26R | Reverse | GCTAATCAAGGTTTTTATTGGTC |
| f-NcSP38F | Forward | ACAGACCTGCTACAAGGTGTCTC |
| f-NcSP38R | Reverse | CTTGAATCCTATTCCAAACTCAGTTG |
| f-NcSP70F | Forward | ATCAGTCAGTCAACATGCATCTG |
| f-NcSP70R | Reverse | CACATTGGATTCCGTGTTTCAAAG |
| f-NcSP75F | Forward | CAACTATTCAAATGAAGAGGCTAAT |
| f-NcSP75R | Reverse | CAAATACACAAGTCAAAAACTAACG |
| RT-PCR |  |  |
| RT-NcSP38F | Forward | AAGACTGGAAGACACGAATG |
| RT-NcSP38R | Reverse | GTTAGCGAATGAAAGACCTC |
| RT-NcSP75F | Forward | CTGGTTCGGACTTTATTCAG |
| RT-NcSP75R | Reverse | ATCGTCTACCACCTCAACAG |
| RT-RpL19F | Forward | GCTTTGGTAAGAGGAAGGGTACTGC |
| RT-RpL19R | Reverse | GCCTCATCTTCCTTCTGGTACGATT |
