# Supplementary material for: Proteome Analysis of Watery Saliva Secreted by Green Rice Leafhopper, Nephotettix cincticeps
Source: PLoS One. 2015 Apr 24;10(4):e0123671. doi: 10.1371/journal.pone.0123671 (PMC4409333; doi:10.1371/journal.pone.0123671)
Supplement: S2 Table — Unknown proteins matched to peptides from salivary gland RNAseq data of N. cincticeps. a The matched unique peptides are shown in S3 Table. b The number of matched ESTs of N. cincticeps, which are derived from the salivary gland (SG). c YES: matched to ESTs from the salivary gland of H. vtripenis. (DOCX) [file pone.0123671.s003.docx]

**S2 Table.** **Unknown proteins identified in watery saliva of *N. cincticeps* by gel-free based nano LC-MSMS.**

| Contig-ID | total ion score | No. of unique peptide matches | MW | pI | No.　of ESTs | SG-EST  (*H.vtripenis*） | Secretion Signal | InterPro ID |
| --- | --- | --- | --- | --- | --- | --- | --- | --- |
| TsukubaH.comp13102_c1_seq1  (TsukubaH.comp13102_c1_seq2) | 7873  (5999) | 36  (11) | 50686.8 | 8.5 | 97 SG  ( 3 SG) | YES | NO | NO |
| TsukubaH.comp13381_c0_seq1 | 873 | 10 | >25847.7 | ~8.0 | 50 SG | NO | YES | NO |
| TsukubaH.comp9384_c0_seq1 | 394 | 16 | 45617.2 | 9.0 | 9 SG | YES | YES | NO |
| TsukubaH.comp13110_c0_seq7 | 358 | 4 | 18403.5 | 5.8 | 3 SG | NO | YES | NO |
| TsukubaH.comp3975_c0_seq1 | 336 | 17 | 101830.3 | 8.6 | 72 SG | YES | YES | NO |
| TsukubaH.comp3976_c0_seq1 | 307 | 4 | 17608.0 | 8.3 | 2 SG | YES | YES | NO |
| TsukubaH.comp13359_c1_seq1 | 259 | 4 | 23122.8 | 4.9 | 48 SG | NO | YES | NO |
| TsukubaH.comp3988_c0_seq1 | 246 | 5 | >7400.3 | ~9.3 | 0 | NO | YES | NO |
| TsukubaH.comp12296_c1_seq1 | 211 | 6 | 17257.5 | 5.1 | 42 SG | NO | YES | NO |
| TsukubaH.comp13516_c0_seq1 | 164 | 3 | 8337.3 | 7.6 | 2 SG | NO | YES | NO |

| TsukubaH.comp7063_c0_seq1 | 153 | 6 | 21304.3 | 7.2 | 17 SG | YES | YES | IPR011038 |
| --- | --- | --- | --- | --- | --- | --- | --- | --- |
| TsukubaH.comp3950_c0_seq1 | 142 | 3 | 5587.2 | 6.2 | 0 | YES | YES | NO |
| TsukubaH.comp3958_c0_seq1 | 140 | 3 | 9364.4 | 5.5 | 15 SG | NO | YES | NO |
| TsukubaH.comp12042_c0_seq1 | 133 | 4 | 18218.6 | 6.4 | 10 SG | NO | NO | NO |
| TsukubaH.comp13442_c0_seq1 | 83 | 6 | 10623.0 | 5.2 | 3 SG | NO | YES | NO |
| TsukubaH.comp9566_c0_seq1 | 81 | 9 | 58516.4 | 8.6 | 66 SG | YES | YES | NO |
| TsukubaH.comp3980_c0_seq1 | 80 | 3 | 21503.4 | 6.9 | 4 SG | NO | YES | NO |

| TsukubaH.comp12630_c0_seq2 | 63 | 2 | 18483.8 | 4.8 | 3 SG | NO | YES | NO |
| --- | --- | --- | --- | --- | --- | --- | --- | --- |

| TsukubaH.comp9557_c0_seq1 | 58 | 2 | 16716.1 | 6.5 | 16 SG | NO | YES | NO |
| --- | --- | --- | --- | --- | --- | --- | --- | --- |
| TsukubaH.comp13484_c0_seq1 | 51 | 2 | 6478.1 | 4.8 | 0 | NO | YES | NO |
| TsukubaH.comp11145_c0_seq1 | 39 | 2 | 1894.5 | 5.9 | 14 SG | YES | YES | NO |
| TsukubaH.comp14063_c0_seq1 | 37 | 3 | 10073.7 | 9.2 | 0 | NO | NO | NO |
| TsukubaH.comp10687_c0_seq1 | 33 | 5 | 64974.2 | 8.7 | 20 SG | YES | NO | NO |

Unknown proteins matched to peptides from salivary gland RNAseq data of *N. cincticeps*. ^a^ The unique matched peptides are shown in S3 Table. ^b^ The number of matched ESTs of *N. cincticeps*, which are derived from the salivary gland (SG).^c^ YES: matched to ESTs from the salivary gland of *H. vtripenis*.
