# Supplementary material for: Proteome Analysis of Watery Saliva Secreted by Green Rice Leafhopper, Nephotettix cincticeps
Source: PLoS One. 2015 Apr 24;10(4):e0123671. doi: 10.1371/journal.pone.0123671 (PMC4409333; doi:10.1371/journal.pone.0123671)
Supplement: S3 Table — (DOCX) [file pone.0123671.s004.docx]

**S3 Table.** **Matched unique peptides detected in the saliva of *N. cincticeps* by gel-free based nano LC-MSMS.**

| Contig-ID | No. of matched unique peptides | Peptide Sequences |
| --- | --- | --- |
| TsukubaH.comp13102_c1_seq1 | 36 | K.RIEYIK.K K.YFDYLK.T K.VDIFNLVK.A K.SLYSVVYK.K -.FYSVYYK.K K.AISGNGAAFEK.K  K.ATETCGLWK.F K.TFYSVYYK.K K.VTDSCGCWK.V K.EESLESYFK R K.ETTEEWFTR.V K.KEESLESYFK.R K.SFFEITESDIK.T K.FATETEEQYIK.R K.FSYFTNIYTTK.V K.VFQSDYLSLLQK.S K.TTTESEELYYSR.I K.KFATETEEQYIK.R K.EAWENEEQYYK.R K.FATETEEQYIK.R R.DFNSLDLWYDIK.Y K.TFFPTQEVYTSVK.S K.CFQSEYFSLIEK.T R.DLYPSLALWTEEK.Y R.ALKPLSGESFEQAVK.R R.VLKPLPGEGFDQSVK.R K.KEAWENEEQYYK.R R.DSFFDTSDLIWCK.K K.EAWENEEQYYKR.I K.SSSSSSSSASSSEEQLK.A R.RDFNSLDLWYDIK.Y K.YIDLVKPYYTALYTK.T K.TEFLSTTESYINTLNILR.R K.IFLIQEATIFSPIWYEAK.Y K.SSSSQSAALSQSADESEEAVTVR.R R.STGTWCISQSISPQSTMQEMGR.G |
| TsukubaH.comp13102_c1_seq2 | 32 | K.YFDYLK.T K.ELTEAEIK.A K.VDIFNLVK.A K.SLYSVVYK.K R.WSWTQPPR.G K.AISGNGAAFEK.K K.TFYSVYYK.K K.VTDSCGCWK.V K.NESEELYYSR.L K.EGESLETYFNR.I K.FATETEEQYIK.R K.KFATETEEQYIK.R K.EAWENEEQYYK.R K.HYSTLALWYDIK.Y K.FATETEEQYIKR.I K.TFFPTQEVYTSVK.S K.CFQSEYFSLIEK.T R.DLYPSLALWTEEK.Y R.ALKPLSGESFEQAVK.R K.KEAWENEEQYYK.R R.DSFFDTSDLIWCK.K K.EAWENEEQYYKR.I -.AGWSAQAAWYHQER.G K.SSSSSSSSASSSEEQLK.A K.TEYTSTPDSYLYILNTVR.K K.IFLIQEATIFSPIWYEAK.Y R.AAAESSSSEEEQLQTGNGLYVK.R K.SSSSQSAALSQSADESEEAVTVR.R R.STGTWCISQSISPQSTMQEMGR.G K.SSSSSAVSSSSSSQEQIIQAGGCGCK.S K.KSSSSSAVSSSSSSQEQIIQAGGCGCK.S K.AVSQNAQQSSESAVVVNDGSGSVVQAQK.S |
| TsukubaH.comp13368_c0_seq1 | 13 | K.CATTLIGK.D K.DVPSGQNYK.T K.TITTYDTFGK.K K.TYIVPFYEK.N K.AEYLGTDGFIK.F K.NGIVGVVTCTDK.K -.IYCSISTMTNK.N K.FTYTTKPADLAK.A K.NGIVGVVTCTDKK.S K.DDVGFYTEGSEGIK.T K.KDDVGFYTEGSEGIK.T K.DGVLVAVTPFYFPVGVK.C K.LGITTWDPAFGSPEECAK.V |
| TsukubaH.comp12770_c0_seq1 | 13 | R.SGFEYACAK.G K.NHLMSTCNA.- R.FWQSVASQFK.N R.NAVIDYVNLLR.Q K.YIDGLMSWLDEK.N  K.GFGISEGPMDDSAIK.V K.VILDLHWSEGYYSGK.N K.GPALITDYNGTPNGFGIGFK.N  K.NINYIAWVWNTWDCAK.G R.DGGSACPGFQFEVAGMQDLVNAVR.S K.NDDGIIFDLFNEPFPDQVINDK.T  R.IPLNEYCWLGLGDFKPEYSGINYR.N K.CWESTIAPVAAQYPVIVGEIGEHDCDHK.Y |
| TsukubaH.comp10542_c0_seq1 | 18 | R.LMLRER.G K.QAINEWVR.R K.LMPYLEER.Q -.TYWLRQSR.N -.TFNAQSYVRK.Y R.FWTDVANMFK.D  R.VPLNEGCWFR.R K.SLANESPPNMIK.F R.FADDAAPITEIMQK.M K.NYDGEPTEFGIGFK.K  K.THTVIVGEIGENDCK.H K.GTTIFDSPNDDATIENMK.K R.QISYLAWTWNNWDCK.N K.NLIMLGGLSFANDLEGWR.E K.DDQGIIFDLFNEPFPER.Q K.GTTIFDSPNDDATIENMKK.W  R. HECPGFEYEVAGMQDLVNAVR.E R.ENLPYDPEDNLAASMHNYNFNSCTNIDCWER.G |
| TsukubaH.comp13506_c0_seq1 | 26 | K.ALSTFFSK.S K.FQVAPVLK.E K.LVTSGLVAR.D K.NMEQLAGLK.S R.YEAVIVVHK.D K.SSPLQSWADLK.G  K.WSPDPEINQR.L K.GPYTPQEYLGK.A K.GVGVEMSCVPAR.D K.CEDLAQAAYSR.D R.CLAVGGGDVAFTK.V  R.AVISGDESRCHK.L K.FFGMAYGSQPAAK.S K.AEDYELLCEAGGR.K K.GVGVEMSCVPARDR.I  K.DDILFALLSSADLYHK.H K.ADWEAVDPEDMYIAAK.R K.ACFANVGEAAGWVIPVHK.L  K.QQYSNLCELCEFPDK.C K.AISEFFSAVNYSEEPFK.C K.SDHKPEDYAYLCPDASK.K  K.ENQADIVSVDPGMAVNALSK.F K.CLGSGEGDVAFTDYDSVLR.H K.LCVPHNAYEACVQMMEQGK.G  R.DIRPGLTCVTRPSLSECLTAIK.E K.GHPNVLFSNVALGLDSVHTGADPLAPHQK.L |
| TsukubaH.comp3969_c0_seq1 | 10 | K.LIEFASEK.T K.YVPDFENNK.D K.YAIIDYQPGK.S K.CTTEQGIDGPDHR.Y R.LFKYLTSAPHFLK.T  R.YILSLDDFTEEEQK.K .TWSYKYVPDFENNK.D .YILSLDDFTEEEQKK.I K.INPPVDDTVFLDTNGEIVSSTVSGGQPK.Y K.DLTSMSLIISPTTSPANSYLLHTIYR.I |
| TsukubaH.comp11865_c0_seq1 | 21 | K.GTIPDIDR.L R.LPVPEIYWK.V K.NWLGVENAVR.R K.EVYLDPLEAR.R R.VLDILLGTDATK.S  K.ACIVAVGQNNVK.L K.LNLQQQLDPLK.E K.EPLILEINETR.D -.VYECASQSSSIK.W K.NTGWPTLNDLDK.G K.LLFACPGPNNNALK.V K.METYYSSHQVYGK.S K.NTHEACGYGDSVGTK.F K.GNEQNQGVLYIPEGK.K R.QQLDFIWPEHVER.G R.EDGVYLGMNPDHAYK.Q R. VVRLHLLYVVADLHQR.E K.GTIPDIDRLPVPEIYWK.V R.LAANEDTTLNVWTGTYGILK.L K.ITDDESESFFPLVEVCHNIEK.M K.LNLQQQLDPLKEPLILEINETR.D |
| TsukubaH.comp13381_c0_seq1 | 10 | K.DYIWEK.D K.IQGTPYTK.A K.LPECYPNGK.A K.EYQIYPISK.E K.MTLWPACATNP.- K.ENHVYFYYR.C  K.ALECTNSLQINNMK.K K.LASGSETTVTYDQDQR.K R.SILTRNHVESSWHFGR.C K.NTLAYISNDVLPDGITSVVGK.K |
| TsukubaH.comp7022_c0_seq1 | 8 | K.IEGFWMSR.S R. NYVYGVGNEHQLR.F K.IEGFWMSRSNPNEETR.N K.GLDQGPTNPQSHIEIYTSK.R  -.SWRGSASCLCIFQYATVGW.- K.VSELSGNDAAALCFVDISQDTK.I -.PIHEYVNLLLDYYFAGVYSLYMK.E  K.NAGTWQHNQLNGQHSGEAFIHQGTDK.D |
| TsukubaH.comp10744_c0_seq1 | 9 | K.TICWPDR.C K.TSSKLFLNIK.T K.DSSTYCELTTGK.C R.LSVGLTAVQLCGALK.I R.APCYPVPECKPNK.C  -.TLTEALRMWCACCVAT.- K.GSCFEETECQPGQCPPR.K K.CASVSCPNGQVCENGECVLDAK.C  K.CVLNTTCTPACSAEEECQLVEVQCIR.A |
| TsukubaH.comp9384_c0_seq1 | 16 | R.SLYALEFK.K K.GESLDVYFK.R K.SLQNEWLVK.V K.NFYSIWYK.K K.ETCDAWLLR.A K.SVTGDCGCWK.L  K.AFFPCGCGGVK.V K.DAYFDFTDYK.W K.EAWETDECFAR.R K.LDSETLESYYSR.I K.SYTWTSTTEGYLK.T  R.ALTPLPGESLDNAIK.R R.RGKPHCTTEGSWK.W K.DFSSLDLWYDRK.Y K.QEKETCDAWLLR.A  K.SSSASSASASESSEYAAVK.V |
| TsukubaH.comp13110_c0_seq7 | 4 | K.LLQSDLLK.C R.ADYCVDQLNQQDYR.A K.IQQLGDISSDVTSVLQQYNGDR.A  K.HEQNMDACITNANFYIPGLQK.T |
| TsukubaH.comp3975_c0_seq1 | 17 | K.INLPESALK.Y K.LDSIIPLLR.E K.SFYEAWYK.K K.TDDFNSYIK.R R.EKTDDFNSYIK.R  K.YGKLDSIIPLLR.E R.ESQQSLQVKPQK.S K.LPIETDVTYQTR.L K.STLEDFTTYVNR.I K.TIQEEIEEIYGK.K  K.QKDEPVENYFSR.V K.EKPMTATSSVCSPNK.K R.CDVLTTVLSVANTLR.K R. LHGALDKDPFQVWR.S  K.STLEDFTTYVNRIK.I R.FGDLPLWYNCEFIDLTK.N R.ITLLQDLYPDLPLWFDEK.Y |
| TsukubaH.comp3976_c0_seq1 | 4 | K.DGVAAWYK.C K.AAFGEPEGAIK.V R.TCSPDVITSIK.L K.SVGITMTTEYKPDC.- |
| TsukubaH.comp13359_c1_seq1 | 4 | K.GIVLIVSR.K R.LLTGQQELGSYEYK.T K.FLLIFDNTFPAIDR.N R.SNNIGVSPVQLSGEYK.F |
| TsukubaH.comp3988_c0_seq1 | 4 | -.EEISKAEQR.L K.NGQYIFVPNSPK.G K.ILQLTPEIPAIPTK.N K.GESSPSQSPSYSGSTTAGSVVAGPNNK.S |
| TsukubaH.comp14402_c0_seq1 | 5 | -.FSILFIQFIK.T K.TFYEQGSLPIQK.I R.SAPSCVEEYIEK.Y R.QSCGGQAIFSASFFRK.T  K.LVGFGLAGFGSYSENNLPTK.T |
| TsukubaH.comp10692_c0_seq1 | 2 | K.EGYAANDFYAK.S K.GTSSADVQSSSTSSVEK.K |
| TsukubaH.comp9291_c0_seq1 | 3 | K.GNNAIAWMSVDNCSGLK.D K.WDYCDHGTCEWHIAASK.V K.VLFTTTPHFLANIQGNPVADTK.H |
| TsukubaH.comp13567_c0_seq1 | 11 | K.VDGIVGSLLIR.Q K.VIEIKQILGGME.- K.TKPIICMILKK.- K.MVGKVAVEEPVTR.V K.DTYPLTAGGYAVTR.I  -.GLSGRSDSTSLLMR.N -.QLPCFTTEMLLR.L R.YQGGPNSPPSAEPDHR.E K.ACNDCLTNMGDCLAR.G  K.LACNNNEKHAMLFLVR.L R.YLHDMHTLPMLCVPSVGPEALWR.T |
| sukubaH.comp12296_c1_seq1 | 6 | R. LIQPLIGK.L -.PSQQALMSLK. L K.ITFCSGNFMIK. C K.AIATISETTACSK.N K.VSLTKPTGFDEFK.A  K.DLAQLPTMSWTLSS.- |
| TsukubaH.comp12976_c0_seq1 | 5 | R.SPGWAWRCT.- K.YLEQVEVTCK.I R.GALTITLTSPAGTK.T R.CTAVFAGAYSGGNSNDEK.V  K.GGKPGSTYSTEPANAANLNTYEGPTK.N |
| TsukubaH.comp7411_c0_seq1 | 3 | R.SMYFCTAFVEQK.- K.YEECASDYFCSTK.A K.AKYEECASDYFCSTK.A |
| TsukubaH.comp13516_c0_seq1 | 3 | K.VYTSYSNER.T K.NGEAHSEIQYQR.G K.YLPQANEASASFLLAR.A |
| TsukubaH.comp4366_c0_seq1 | 6 | R.DDLFNTNASIVR.D K.FFSTPILLGPNGLQK.N K.VAVCGASGGIGQPLSLLLK.E R.NQTVCPIQDFSGVHLAPGG.- K.ALIGIISNPVNSTVPIAAEVLK.K -.MSLSLEVTPESQSSPSSLSALLLYPSR.R |
| TsukubaH.comp7063_c0_seq1 | 6 | K.LQDYIDK.D K.MNIIDYNK.E R.LAVPLMQVEQSLAK.C R.TFLFYKMSMMVQGFVFTIK.L  R.TCYISYVGLMFLAYVSYLGM.- R.VLYYCVDSPLANPDIYNVQIATVEQVPANLASVLAAGK.T |
| TsukubaH.comp11079_c0_seq1 | 7 | R.VGTLQRR.S R.VSTAMVNLERT.- K.MKETAEAYLGK.T K.DTILNGQDGDIK.G R.TTPSYVAFTDTER.L  R.IINEPTAAAIAYGLDK.K R.KCTLVDLLDGGSRPSSSTR.G |
| TsukubaH.comp3950_c0_seq1 | 2 | K.SSSTSLLTNTNVYTPK.- K.SCNDIGVCNSYDGYIK.K |
| TsukubaH.comp3958_c0_seq1 | 3 | K.EVEMIAIIK.N R.VSQLSFSKPADTQSYPGAEPPR.S K.NEETSSFYTETEEESSYQAEQK.A |
| TsukubaH.comp13568_c0_seq1 | 7 | R.LTDTPLAVFK.V R. IPGPSVEVCLGDK.I -.TCNNRSYVHPR.H K.DLYCSSQDLSSCK.R  R.LQTGELYLQEVHAAV.- K.TYNANHPFHLHGYAFRVVGMDR.L K.HNVTYISSDGYDFDPVVADSLVSYAGER.W |
| TsukubaH.comp11772_c0_seq1 | 6 | R.SIQFPVSR.V R.LAMEPVVKS.- K.LTMSSTTLTSR.A K.VLYYIDTPTR.R R.VDAFDYDLTTGSIANR.R  R.VSSVAWGGAVLDTLYVTTIK.H |
| TsukubaH.comp12042_c0_seq1 | 4 | K.AVAIIWESTVCEK.D K.YCFDESCSVSSHSSLPR.T R.QQNFDLHYLVRACCFITR.-  K.AFLFLEDCAQIVDVAIRDEETENK.H |
| TsukubaH.comp12022_c0_seq1 | 9 | -.NGTYLPVA.- -.LWTTKLEK.Q R.QTTIPSTIGEEK.E R.VLMSAKNFCIVG.- R.NLMMLTHKYNR.Y  K.FASFVDSENGAVAK.K K.VFCGHEYTYANLK.F K.NEPQAVFTGDTLFIAGCGR.F  R.EAAVVDPVEPEHVLAELQGK.D |
| TsukubaH.comp3954_c0_seq1 | 4 | R.WPLEYVK.F R.VLPDITQPSSYR.E K.EDPSLRPQLIGGPLK.D K.FDHPIQSPIDLLTSK.A |
| TsukubaH.comp12518_c0_seq1 | 4 | -.TRFHLFLSL.- K.VDTEDTTAAKPETVEEK.S R.TESSSVVAEEVPIGQSPVK.Q R.TVDSEEILPFTPDIENGTK.K |
| TsukubaH.comp13792_c0_seq1 | 3 | K.IGINGFGR.I K.AEGDFLVVNGNK.I R.VPVPNVSVVDLTVR.L |
| TsukubaH.comp10065_c0_seq3 | 4 | R.VESHFVTSTISMYR.S -.SQTSLECLCFGLFDR.M K.ANGMSLPAMVHLSTSAYRDASHPL.-  K.AATSENANTQSTQETGSDGSAAPIPVSK.E |
| TsukubaH.comp10607_c0_seq1 | 4 | R.AEWGSKSEER.F K.NDLVVLEDFK.I K.LFAITKMENVMMNLGR.S -.QPSIRIFLLFFISIASPPHMTCALMS.- |
| TsukubaH.comp13482_c0_seq1 | 9 | -.LFGFRGK.C K.FVSEELLK.T K.LQMLSLMRT.- K.IDVVTPGLIIK.T K.QRQQNEAHGR.G K.TESDFESEIDK.G  K.EFPTEISLENDIK.G -.FTVFDINVLYCR.N R.MPQNGHTIMTVAVAQDSKK.L |
| TsukubaH.comp11730_c0_seq1 | 2 | R.AIGGGLSGSPADSR.Q K.VLNTASGTTDGQTISPSDK.S |
| TsukubaH.comp13442_c0_seq1 | 2 | R.GVNCEVIDELSCQK.G K.FGQFDVIIGGEHQEVTLPC.- |
| TsukubaH.comp12456_c0_seq1 | 4 | K.LYNLEVIF.- -.LNLSNSTGFI.- K.ILNAGLVTAEELK.K K.LEQGPSTTVTISR.D |
| TsukubaH.comp9566_c0_seq1 | 9 | R.IEFIFNK.V -.LMLALQLGSH.- K.ESVDEWIAR.I R.DFFGEYNPK.M R.LDGESFDHYR.K  R.LNENFLHQMK.- K.VYEGEEPQQYIER.I R.TNPVFVSMMEHYYR.E R.IDYLFDYVYPELDIR.T |
| TsukubaH.comp3980_c0_seq1 | 3 | K.EYDIEGEVCSK.E K.SEPVWNGGVTLLK.T -.PFHVNDNDNYTHLGK.R |
| TsukubaH.comp13746_c0_seq1 | 5 | R.ARGHVLHK.T -.TPSGLTIIK.V K.FTWASEELLK.Q R.FLLGTTNPQDSNPGTIR.G K.STSLHYIQPFPDHWDHRAR.G |
| TsukubaH.comp12767_c0_seq1 | 3 | R.LPAAARGR.R K.ETQDHGDLQR.T R.GPCLDGPADAVSDDPSSLR.R |
| TsukubaH.comp3925_c0_seq2 | 6 | R.IQSPIDIILSK.V K.IPYMEFGPLPK.K K.SVPGLENLNNLK.I R.TTSLQTSEHSTTKK.A  R.ALYHEEGSSPIVENK.R R.CVESASSLMEITLEILMTLCLPMTK.R |
| TsukubaH.comp4557_c0_seq1 | 3 | K.FLFQNYNNNK.Y -.PTVIASSLREFR.- K.VVIAYEPVWAIGTGK.T |
| TsukubaH.comp13820_c0_seq1 | 6 | R.AEPLGDSLWFK.L K.TGEWVIPLTLDEAK.Q -.TSYMGHAWGSSLVQR.M K.VTSPTPTSWPPSYMVK.K  R.VQSNVYNQGSGLEITREIVLSV.- R.QFGGIGLHMYMTESPELIKCAC.- |
| TsukubaH.comp12630_c0_seq2 | 2 | R.DNSASQSMFLVHTVR.G R.TSSEEPGITYTLADGTTVTAK.A |
| TsukubaH.comp12438_c0_seq2 | 2 | K.YLHTTTESQNQMK.S K.TITLEVEPSDTIENVK.A |
| TsukubaH.comp14120_c0_seq1 | 2 | K.NVLAAGPCHEISPVLK.A K.EAVTAAVAQLNEAAETLHHAGSDLK.Q |
| TsukubaH.comp9557_c0_seq1 | 2 | K.WQCTESLPLREK.L K.SGLCETDYFDPSGNVPK.A |
| TsukubaH.comp13035_c0_seq1 | 5 | K.AGLEVTQQK.E K.IDIGMDVAASEFYK.D K.VNQIGSVTESIEAHLLAK.K R.SGETEDTFIADLVVGLSTGQIK.T  -.QTWVCSEPLSHPEPPLVFMR.L |
| TsukubaH.comp13484_c0_seq1 | 2 | R.SRHIYAIMR.R R.HCTHTGLHCFSDDDDDLSCK.L |
| TsukubaH.comp13308_c1_seq1 | 3 | R.TVVVHADPDDLGK.G K.GTIYFEQSDDGAPVK.V R.SSACNCTLESLAVLYGHS.- |
| TsukubaH.comp3848_c0_seq1 | 3 | K.LPQEIIEVTYDGK.H K.MDKLEIVPDVIDK.L -.AIQLGETAIKLNGMTMCLYYINNWEAK.N |
| TsukubaH.comp11046_c0_seq1 | 3 | R.ASEQIPGLPR.L -.LYAIRHSPATVVL.- K.VPEESDLVDVGEHADAEVR.G |
| TsukubaH.comp11145_c0_seq1 | 2 | R. VIVEYNDPK.K K.ENLVISYQHEEASGYIK.E |
| TsukubaH.comp14063_c0_seq1 | 3 | R.FVVDQSGVNTR.D -.DWLTLIATMYK.V R.AASTLETPGSHATPPFGSSRTSSPMSTSPR.H |
| TsukubaH.comp10687_c0_seq1 | 5 | R.SSTLSITPKR.L K.HFFEIYYK.K K.EGISTEEYIK.R K.TEETEESYFQR.L K.SSSQSSSSSESSEESVVVK.S |
| TsukubaH.comp13467_c0_seq1 | 3 | -.NGRIVTIPSGSPR.G K.NYNQLLGLDEIR.I R.MLPPYEHLHHYSRK.H |
| TsukubaH.comp11983_c0_seq4 | 5 | K.GMAFTLEER.Q K.YIYLMGLQDR.N R.NMMTSLMSSCK.L -.FLGQGYVQGMAGR.-  R.FLSENVEQLMPIVYTPTVGLACQK.Y |
| TsukubaH.comp10419_c0_seq1 | 3 | -.PYIGIVRK.F -.LALWTEEK.N -.ASGWTPAGCLPP.- |
| TsukubaH.comp3952_c0_seq1 | 2 | R.FMLNGAVQSER.E K.LYDGQWESCNR.R |
| TsukubaH.comp10152_c0_seq1 | 5 | R.RLTSTPPR.I K.VSSTLSGLEGELK.G -.PCMERPQRTR.V R.VEMMNSPAGSRLLK.L R.LISMQMGGDLGQVYR.R |
| sukubaH.comp7644_c0_seq1 | 3 | K.EQLQWRR.Q K.NASNMVNDAVDQGTATMEK.A K.SVVGAIGGGIETAGNVGAAGMSIAQGGANLGIK.T |
| TsukubaH.comp3946_c0_seq1 | 2 | K.VTETVLAAVYK.A -.FLNSYIKIFK.T |
| TsukubaH.comp13836_c0_seq1 | 2 | K.IGPLGLSPK.K R.GGSLSALITR.E |
| TsukubaH.comp4079_c0_seq1 | 3 | R.NAQYDQFLEDLQK.A K.RGCSLLPLPTEQNK.M K.QNYNYNCVDVFSFLIK.A |
