# Supplementary material for: Proteome Analysis of Watery Saliva Secreted by Green Rice Leafhopper, Nephotettix cincticeps
Source: PLoS One. 2015 Apr 24;10(4):e0123671. doi: 10.1371/journal.pone.0123671 (PMC4409333; doi:10.1371/journal.pone.0123671)
Supplement: S4 Table — NcSP84 (AB618633) and NcSP75 (LC009515) were identified only by in-gel method (see Table 1). a The unique matched peptides are shown in S3 Table. b YES: matched to ESTs from the salivary gland of H. vtripenis. (DOCX) [file pone.0123671.s005.docx]

**S4 Table. Proteins identified in watery saliva of *N. cincticeps* by both in-gel method and gel-free based nano LC-MSMS.**

| In-gel method | Accession No. | Gel-free method | Contig-ID | Total ion score | No. of unique peptide matches ^a^ | SG-EST ^b^  (*H.vtripenis*) |
| --- | --- | --- | --- | --- | --- | --- |
| NcSP70 | LC009513 | Identified | TsukubaH.comp13506_c0_seq1 | 1769 | 26 | NO |
| NcSP38 | LC009514 | Identified | TsukubaH.comp10542_c0_seq1 | 1826 | 18 | YES |
| NcSP26 | LC009515 | Identified | TsukubaH.comp13368_c0_seq1 | 4037 | 13 | NO |
| NcSP23 | LC009516 | Identified | TsukubaH.comp3969_c0_seq1 | 1367 | 10 | YES |
| NcSP22 | LC009517 | Identified | TsukubaH.comp7022_c0_seq1 | 569 | 8 | NO |
| NcSP19 | LC009518 | Identified | TsukubaH.comp3976_c0_seq1 | 229 | 4 | YES |
| NcSP16 | LC009519 | Identified | TsukubaH.comp9291_c0_seq1 | 228 | 3 | NO |

NcSP84 (AB618633) and NcSP75 (LC009515) were identified only by in-gel method (see Table 1). ^a^ The unique matched peptides are shown in S4 Table. ^b^ YES: matched to ESTs from the salivary gland of *H.vtripenis*.
